# Psychosocial factors associated with the trajectories of interparental conflict for Australian fathers of autistic children: A longitudinal study across 10 years of child development

**DOI:** 10.1177/13623613251316014

**Published:** 2025-02-08

**Authors:** Monique Seymour, Grace McMahon, Ali Fogarty, Bridget O’Connor, Mark Feinberg, Rob Hock, Rebecca Giallo

**Affiliations:** 1 Centre for Social and Early Emotional Development (SEED), School of Psychology, Faculty of Health, Deakin University, Australia; 2 Intergenerational Health, Population Health, Murdoch Children’s Research Institute, Australia; 3 Mental Health Department, Royal Children’s Hospital, Australia; 4 Department of Paediatrics, The University of Melbourne, Australia; 5 Neurodisability and Rehabilitation, Murdoch Children’s Research Institute, Australia; 6 The Pennsylvania State University, USA; 7 College of Social Work, University of South Carolina, USA

**Keywords:** autism, fathers, interparental conflict, psychosocial factors, trajectory

## Abstract

Limited research exists on fathers’ experiences of interparental conflict (IPC) in families with autistic children. We aimed to identify: (1) the extent to which these fathers report IPC across 10 years of child development (4–14 years) and how this compares to fathers of non-autistic children; (2) distinct trajectories of IPC for fathers of autistic children and (3) factors associated with trajectories of IPC among fathers of autistic children. This is a retrospective study using a national dataset. Participants were 281 fathers of autistic children and 7046 fathers of non-autistic children. Although small effect sizes, fathers of autistic children, on average, reported significantly higher IPC when their child was 4–5, 6–7, 8–9 and 10–12 years of age, compared to fathers of non-autistic children. For fathers of autistic children, longitudinal latent class analysis revealed three profiles reflecting ‘*low and stable*’, ‘*moderate and stable*’ and ‘*persistently elevated*’ levels of IPC over time. Regression analysis revealed a range of predictors (e.g. co-parenting support, father age) for fathers of autistic children experiencing ‘*moderate and stable*’ and ‘*persistently elevated*’ IPC as compared to fathers who experienced low levels of IPC. There is a significant portion of fathers of autistic children who experience ongoing and heightened IPC, highlighting the need for targeted support.

**Lay Abstract:**

Not much is known about how fathers experience conflict with their partners (either verbal or physical) while raising an autistic child. This study focused on understanding these experiences over 10 years, following children from the age of 4 to 14 years. The study had two main goals: (1) to track how fathers experience conflict with their partners over this time and identify different patterns to these experiences; and (2) to find psychosocial factors in early childhood that might impact these patterns. The study included 281 fathers of autistic children and 7046 fathers of non-autistic children who took part in ‘*Growing Up in Australia: Longitudinal Study of Australian Children*’. Using a statistical method to group fathers based on the partner conflict they reported over the 10 years, results showed that there were three groups: (1) ‘*low and stable*’, (2) ‘*moderate and stable*’ and (3) ‘*persistently elevated*’ experiences of partner conflict. Additional analysis showed that fathers’ confidence in their parenting, perceived support from their partners and concerns about their child’s language skills were associated with ongoing moderate levels of partner conflict. On the other hand, fathers who were older, had lower perceived support from their partners, partners experiencing psychological distress and higher parent-reported child social functioning were more likely to experience consistently high levels of conflict over time. In our study, we described different levels of conflict with their partners reported by fathers of autistic children. We also identified some of the factors that were associated with different levels of conflict. These might be used to inform interventions to reduce parental conflict in the future.

## Introduction

Parenting an autistic child places additional stresses and strains on parents, including managing core traits of autism (e.g. communication differences, reciprocal social interactions, sensory sensitivities, repetitive behaviours, specialized interests), co-existing symptoms and conditions (e.g. attention-deficit hyperactivity disorder, epilepsy); navigating services and resources for their child; advocating for their child; lifestyle accommodations and adjustments; along with coping with financial strain and social isolation (Brobst et al., 2009; Hock et al., 2012; Pozo & Sarriá, 2015; Saini et al., 2015). Parents of autistic children consistently report experiencing high levels of parenting stress and mental health difficulties (e.g. depression, anxiety) and significantly more so than parents of non-autistic children (Hayes & Watson, 2013; Papadopoulos et al., 2019; Seymour et al., 2017). Spillover theory (Repetti, 1987) suggests that mood and affect (e.g. parent mental health) and family stressors (e.g. parenting stress, managing additional child and family demands) can have significant impacts on the couple’s relationship (Sanders & Keown, 2022) including interparental conflict (IPC; Piro-Gambetti et al., 2023).

IPC refers to commonly experienced forms of conflict or hostile behaviours between parents, including verbal (e.g. disagreements, arguments, anger, hostility) and physical conflict (e.g. pushing, shoving; van Eldik et al., 2020; Westrupp et al., 2015). IPC is an important aspect of the couple’s relationship to consider, having been identified as a risk factor for poor relationship quality (Bühler et al., 2021) and intimate partner violence (which includes any behaviour within a current or former intimate relationship that causes physical, sexual and/or psychological harm, including coercive control; van Eldik et al., 2020). In community samples, previous research has also documented associations between frequent and more intense IPC and poor outcomes for children (e.g. reduced cognitive appraisal, emotional-behavioural problems) across the lifespan (Giallo, Seymour, Fogarty, et al., 2022; Kopystynska et al., 2022; van Eldik et al., 2020). Even relatively low to moderate levels of IPC have been found to be consistently associated with poor concurrent and later child and parent mental health outcomes, parenting behaviours and parent-child relationships (Giallo, Seymour, Fogarty, et al., 2022; Kopystynska et al., 2022; Rhoades, 2008; van Eldik et al., 2020; Westrupp et al., 2015).

Much of the literature on the couple’s relationship between parents of autistic children focuses broadly on satisfaction and adjustment (Saini et al., 2015). Parents of autistic children report significant pressure on the couple’s relationship (Hock et al., 2012). Compared to mothers of non-autistic children, mothers of autistic children report higher rates of divorce/separation (Hartley et al., 2010; Saini et al., 2015) and lower couple satisfaction and adjustment (Brobst et al., 2009; Higgins et al., 2005; Saini et al., 2015). Emerging IPC research suggests that both mothers and fathers of autistic children report more frequent, severe and unresolved couple conflict than parents of non-autistic children (Hartley et al., 2017; Papp & Hartley, 2019). This conflict is likely due to high child-related challenges (e.g. co-occurring conditions, externalizing behaviour problems, service access) associated with autism, but not autism per se (Hartley et al., 2017; Papp & Hartley, 2019).

Cross-sectional research on fathers of autistic children has found that these fathers report more frequent and severe conflict with their partners than fathers of non-autistic children (*M* child age = 7.88 years; Hartley et al., 2017). From the same sample, a subsequent analysis revealed that over a 14-day period, fathers (*n* = 169) of autistic children reported higher daily IPC rates than fathers of non-autistic children (Papp & Hartley, 2019). These fathers were also more likely to identify their children to be the focus of conflict (Papp & Hartley, 2019). Despite fathers of autistic children facing more severe and ongoing relationship difficulties than fathers of non-autistic children (Saini et al.), limited evidence exists on the long-term experiences of IPC among fathers of autistic children.

From the general family research literature, IPC can persist throughout childhood for some families. In a longitudinal population-based sample of 4136 fathers of children (aged 6–12 months through to 10–11 years; no childhood medical/psychiatric diagnoses identified), two distinct trajectories of IPC were identified over 10 years of child development (Giallo, Seymour, Fogarty, et al., 2022). While most fathers (94%) experienced relatively low levels of IPC over time, 6% of fathers were assigned to a group characterized by higher IPC mean scores which increased over time.

To date, research on IPC among parents of autistic children has largely focused on small cross-sectional samples, and recruitment has occurred predominately through autism service providers in the United States, thus limiting generalizability to families who cannot access supports or wider international contexts. To our knowledge no longitudinal study has explored experiences of IPC among fathers of autistic children within a large community cohort. Autistic children, and their families, have unique needs and challenges which change over time (Gray, 2006). It is important to gain an understanding of the experiences of IPC across child development, specifically for fathers of autistic children, as this will enable timely and targeted support.

It is also essential to explore individual, family, child and sociodemographic risk factors associated with experiences of IPC for fathers of autistic children as this will allow for the identification of modifiable factors, highlighting appropriate avenues of support to help reduce the direct and indirect impacts of IPC. The Bioecological (Bronfenbrenner & Morris, 2006) and the Lifecourse Health Development (Halfon & Hochstein, 2002) frameworks are useful theoretical models in guiding potential factors associated with IPC. These models emphasize complex interactions between the individual and multiple domains (e.g. child, family and society) and how they impact health and development over time. Little to no research has focused on factors associated with IPC for families of autistic children (Saini et al., 2015). Yet, in Saini et al.’s (2015) scoping review of couple’s relationships among parents of autistic children, a range of factors associated with couple’s relationship satisfaction were identified, providing some guidance on what may impact IPC. Individual parental factors worth considering included mental health, self-efficacy and age. Child factors included gender, behaviour and social functioning, while social-environment factors included life stress, socio-economic status, nationality, employment, education and income (Saini et al., 2015). Guided by the broader literature on the couple’s relationship in parents of autistic children, additional factors likely to impact IPC in this group of fathers include child communication, the coparenting relationships, partner mental health and number of children in the household (Chan & Leung, 2020; Langley et al., 2017; Piro-Gambetti et al., 2024).

The overall objective of the current study was to explore experiences of IPC across 10 years of child development for fathers of autistic children. There were three aims: (1) to identify the extent to which fathers report IPC at six timepoints, from when their children were aged 4–5 years to 14–15 years and to explore differences in reports compared to fathers of non-autistic children; (2) to explore distinct trajectories of IPC across 10 years of child development for fathers with autistic children and (3) to identify individual, child, family and socio-demographic factors associated with trajectories of IPC among fathers of autistic children. Although the study was novel and exploratory, driven by ecological and life course frameworks that do not suggest specific pathways or mechanisms, we generated exploratory hypotheses. First, we expected to identify at least two distinct patterns of IPC, one of which would represent high IPC over time. Second, we also expected that contextual factors that are potentially stressful for families (e.g. parent mental health problems, child behaviour difficulties) would be associated with trajectories of high IPC.

## Methodology

### Study design and participants

Data were drawn from the *Growing Up in Australia: the Longitudinal Study of Australian Children* (LSAC), a nationally representative study following the development of children and their families. Ethics approval was granted by the Australian Institute of Family Studies Ethics Committee, and comprehensive study information has been published previously (Soloff et al., 2005). Children were selected from Australia’s universal health database (Medicare) in 2004. There are two cohorts of children: (1) the Baby cohort (B-cohort) when children were recruited in the first year of life; and (2) the Kindergarten cohort (K-cohort) when children were recruited at age 4–5 years. Data are collected biennially, and there are nine waves of data currently available.

Data for this study were drawn from the B- and K-cohorts at six timepoints: when child ages overlap at 4–5 years (B-cohort wave 3, K-cohort wave 1), 6–7 years (B-cohort wave 4, K-cohort wave 2), 8–9 years (B-cohort wave 5, K-cohort wave 3), 10–11 years (B-cohort wave 6, K-cohort wave 4), 12–13 years (B-cohort wave 7, K-cohort wave 5) and 14–15 years (B-cohort wave 8, K-cohort wave 6). Given that the preliminary data analysis failed to reveal significant cohort effects (Australian Institute of Family Studies, 2011), data from the two cohorts were combined.

Child diagnosis of autism was provided by a single self-reported item (i.e. ‘Does study child have any of these conditions: Autism, Asperger’s, or other autism spectrum?’) by the child’s primary caregiver (~93% mothers), at any timepoint (child ages 6–7, 8–9, 10–11, 12–13 and 14–15 years). Information on autism diagnosis was not collected at the child age 4–5 years, as such an autism diagnosis was implied at this timepoint as the mean age of diagnosis for the current sample was 48.37 months (4.03 years). Generally, children who no longer meet diagnostic criteria for autism continue to show delays in one or more neurodevelopment areas (Benedetto et al., 2021). Autism has been found to be a relatively stable diagnosis (Woolfenden et al., 2012).

A total of 381 autistic children were identified, with data available for 307 (male and biological, adoptive or step) fathers across the six timepoints of interest. Of these fathers, 26 did not have IPC data at any timepoint, which meant the final sample comprised 281 fathers of autistic children (B-cohort *n* = 176; K-cohort *n* = 105). Of the non-autistic children, 7842 were identified, and data were available for 7046 fathers (*n* = 796 excluded due to missing IPC data). Demographic characteristics of the final sample are provided in Table 1. Independent sample *t* tests and chi-square (χ^2^) analyses conducted on continuous and categorical variables revealed that fathers of autistic children were significantly more likely to report that they were born in Australia (*p* = .001), were experiencing financial hardship (*p* = .033) and had less children in the household (*p* = .003) than fathers of non-autistic children.

**Table 1.** Child and father demographic characteristics at child age 4–5 years.

Variable	Autistic children (*n* = 368), *n* (%)	Non-autistic children (*n* = 7842), *n* (%)
Gender, male	281 (73.8)	3930 (50.1)
Age (years), *M* (*SD*)	4.18 (0.39)	4.21 (0.41)
Age of diagnosis (months), *M* (*SD*)	75.51 (40.44)	-
Primary caregiver gender, female	356 (93.4)	7619 (97.2)
Possible intellectual disability		
Yes	159 (45.2)	2543 (35.4)
Missing	16 (4.4)	664 (8.5)
Medical conditions		
Sight problems	6 (2)	49 (0.5)
Hearing problems	5 (1.6)	119 (1.3)
Speech problems	93 (30.3)	507 (5.6)
Blackouts	6 (2)	26 (0.3)
Learning difficulty	63 (20.5)	99 (1.1)
Difficulty with use of arms/ fingers	8 (2.6)	10 (0.1)
Difficulty with use of legs/feet	6 (2)	14 (0.2)
Other physical condition	10 (3.3)	26 (0.3)
Disfigurement	2 (0.7)	15 (0.2)
Breathing difficulties	9 (2.9)	100 (1.1)
Chronic pain	2 (0.7)	4 (0)
Nervous condition	9 (2.9)	15 (0.2)
Head injury	5 (1.6)	10 (0.1)
	Fathers (*n* = 281)	Fathers (*n* = 7046)
Age (years), *M* (*SD*)	37.35 (5.98)	37.90 (5.87)
Australia born	236 (84.0)*	5327 (75.6)*
Aboriginal or Torres Strait Islander	3 (1.1)	95 (1.3)
Remoteness, major city	182 (64.8)	4625 (65.6)
Experienced financial hardship	78 (25.4)*	1624 (20.8)*
SEIFA	1011.85 (59.77)	1016.91 (59.70)
Education, year 12 or above	165 (58.7)	4024 (57.1)
Full-time employed	273 (97.2)	6674 (94.7)
Father relationship to study child		
Biological	274 (97.5)	6900 (97.9)
Adoptive	1 (0.4)	12 (0.2)
Step-parent	6 (2.1)	134 (1.9)
Parents are partners	278 (98.9)	7019 (99.6)
Two or more children	246 (87.5)*	6482 (92.0)*

SEIFA = Socio-economic Index For Areas^a^, based on population census data. Mean of 1000, lower scores indicative of more disadvantaged area.

a Australian Bureau of Statistics (2013).

* Significant difference.

### Measures

*IPC* was measured at all waves using an adapted version of the IPC subscale from the Co-Parental Communication Scale (Ahrons, 1981; Gray & Sanson, 2005). Fathers were asked to indicate how often a range of verbal conflict behaviours (four items; e.g. disagreements, arguments, stressed conversations, expressions of anger and hostility) and physical conflict behaviour (one item; i.e. arguments that involve physical confrontation or aggression including pushing, hitting, kicking or shoving) occurred with their partner on a 5-point scale (ranging from 1 = never to 5 = always; theoretical range = 5–25). The five items were summed, with higher scores indicating higher frequency of conflict. The scale has good internal consistency and good construct validity (Giallo, Seymour, Treyvaud, et al., 2022) and has been used in community samples of parents (Giallo, Seymour, Fogarty, et al., 2022; Westrupp et al., 2015). To estimate the proportion of fathers reporting high verbal and physical conflict, high verbal conflict was defined as endorsement of ‘often’ or ‘always’ on at least one verbal IPC item, and high physical conflict was defined as endorsement of ‘sometimes’, ‘often’ or ‘always’ on the physical item (Giallo, Seymour, Fogarty, et al., 2022; Westrupp et al., 2015). The total scale score was used in the analysis estimating the IPC trajectories and the identification of risk factors. Cronbach’s α ranged from .75 to .77 over the timepoints of interest.

#### Predictor variables

Guided by the Bioecological (Bronfenbrenner & Morris, 2006) and Lifecourse Health Development (Halfon & Hochstein, 2002) models and factors linked with couple satisfaction from the study by Saini et al. (2015), several factors that may be associated with fathers’ IPC were explored (Table 1). All predictor variables were assessed at the earliest timepoint (i.e. child aged 4–5 years). The LSAC used standardized measures where possible.

**Table 2.** Potential predictor variables, as measured at child age 4–5 years.

Construct	Measure (source)	Additional information
*Individual characteristics*		
Age		Age of father at last birthday (years)
Father psychological distress	Kessler-6^a^	Six items reporting on feelings of nervousness, hopelessness, restlessness, sadness and worthlessness. Rated from 0 = None of the time to 4 = All or most of the time. Higher scores indicate greater psychological distress. Cronbach’s α = .82.
Father parenting self-efficacy	Early Childhood Longitudinal Study^b^	A single item (‘Overall, as a parent, do you feel that you are . . .’) rated from 1 = Not very good at being a parent to 5 = A very good parent. Higher scores indicated better self-rated parenting self-efficacy.
*Interpersonal characteristics*		
Child gender		Female = 0; Male = 1.
Child behaviour	Total Difficulties of the Strengths and Difficulties Questionnaire^c^	20 items rated by primary caregiver, for example, ‘Restless, overactive, cannot stay still for long’ and ‘Often seemed worried’. Rating scale from 0 = Not true to 2 = Certainly true; higher scores reflect more emotional or behaviour difficulties for the child. Cronbach’s α = .77.
Child social functioning	Adapted from the Paediatric Quality of Life (PedsQL) 4.0 Parent Report for Toddlers: Social functioning^d^	Five items rated by primary caregiver, for example, ‘How often would you say that this child has a problem with . . . Getting teased by other kids; socialising with other kids?’ Rated on a 5-point scale, from 1 = Never to 5 = Almost always. Scored by deriving the mean of items and reverse coded, so that higher scores indicate better social functioning. Cronbach’s α = .77.
Child receptive language ability	Parents Evaluation of Developmental Status PEDS^e^	Single dichotomous item completed by primary caregiver. ‘Do you have concerns about . . . how child understands what you say?’ 1 = Yes; 2 = No.
Child expressive language ability	Parents Evaluation of Developmental Status PEDS^e^	Single dichotomous item completed by primary caregiver. ‘Do you have concerns about . . . how child talks and makes speech sounds?’ 1 = Yes; 2 = No.
Mothers’ mental health^f^	Kessler-6	As above for fathers’ psychological distress. Cronbach’s α = .84.
Co-parenting support	Subscale from Quality of Co-parental Interactions Scale^g^	Mean of three items (e.g. ‘Do you feel your partner understands and is supportive of your needs as a parent?’) rated on a 5-point scale (from 1 = Never to 5 = Always). A higher score indicates greater support. Cronbach’s α = .99.
Number of children		Number of children in home; continuous
*Social environment characteristics*		
Educational attainment		Completed year 12 or equivalent = 0; did not complete = 1.
Employment status		Part-time/Full-time = 0; not in paid employment = 1.
Language		Non-English speaking = 0; English = 1.
Aboriginal or Torres Strait Islander		Not indigenous = 0; Yes, Aboriginal and/or Torres Strait Islander = 1.
Socio-economic position	Socio-Economic Index for Areas – Index of Relative Disadvantage SEIFA^h^	Based on 2011 population census data, including income, educational attainment and employment. Based on a mean of 1000 (SD = 100). Lower scores indicative of an area relatively disadvantaged, used as a continuous measure.
Family income		Yearly income ⩽ $25,999 = 1; $26,000–$51,999 = 2; $52,000–$103,999 = 3; ⩾$104,000 = 4.
Remoteness		Regional or remote area = 0; major city = 1.
Stressful life events	Center for Mental Health Research^i^	Number of life stressors (e.g. parent, partner or child death; illness, injury or assault; major financial crisis; legal problems; relationship separation). No stressors = 0; one or more events = 1.

a Kessler et al. (2002).

b The National Center for Education Statistics (2000).

c Goodman et al. (2000).

d Varni et al. (2001).

e Glascoe (2000).

f Chan and Leung (2020).

g Ahrons (1981).

h Australian Bureau of Statistics (2013).

i Center for Mental Health Research (2005).

### Community involvement

Community members were not involved in any aspect of the research process.

### Data analysis and screening

Descriptive statistics for sample demographics and IPC at each timepoint (Aims 1 and 2) were conducted in SPSS version 29 (IBM Corp., 2021). Independent sample *t* tests and chi-square (χ^2^) analyses were conducted on continuous (total IPC) and categorical (IPC Verbal subscale) variables, to explore differences between fathers of autistic children compared to fathers of non-autistic children. No comparisons were made on the IPC Physical subscale due to the small sample size. Findings are discussed with and without Bonferroni adjustment for multiple comparisons as both are inherent with limitations. While adjustments are recommended in large bodies of data to avoid rejecting the null hypothesis too readily, Bonferroni adjustment is a conservative method for controlling for Type I error rate, and it can be overly stringent. As a result, the adjustment may increase the risk of Type II errors (i.e. failing to detect a true difference; Rothman, 1990). Cohen’s *d* was used to measure effect size (0.3 = small, 0.5 = medium, 0.8 = large) for the complete cases analysis. Next, longitudinal latent class analysis (LLCA) was conducted in MPlus version 8.7 (Muthén & Muthén, 2023) to identify the distinct trajectories of IPC over six timepoints for fathers of autistic children (Aim 3), and separately for fathers of non-autistic children to allow for descriptive comparisons. LLCA involved beginning with a one-class model and subsequently fitting models with increasing numbers of classes to identify the smallest number of classes that best fit the associations in the data. Given this is a data-driven approach, a range of criteria were considered when determining the best-fitting model (Weller et al., 2020). When comparing models, lower values for the Likelihood Ratio Statistic (L2), Akaike Information Criterion (AIC) and the Bayesian Information Criterion (BIC) indicated better fitting models. Entropy value was also considered to determine the accuracy with which the models classified fathers into their most likely class, with values > .80 indicating good classification accuracy. The Vuong-Lo-Mendell-Rubin Likelihood Ratio Test was used to examine the improvement between neighbouring class models (i.e. comparison of two vs three classes), with values < .05 indicating a statistically significant improvement in model fit when an additional class is added. Finally, clinical meaningfulness and class sizes were considered when making a final decision about model solutions. Statistical and graphical measures of normality revealed that distributions for IPC at each wave were generally slightly positively skewed. Therefore, robust maximum likelihood estimation was used to adjust the fit indices and parameter estimates to account for non-normality when conducting the latent class analysis.

To address Aim 4, the class membership of IPC for fathers of autistic children was recorded and used in logistic regression analyses to identify psychosocial factors associated with trajectories of IPC. Univariate and multivariate logistic regression models were estimated in SPSS, where results are presented as odds ratios with 95% confidence intervals. Prior to conducting the regression analysis, bivariate correlations among study variables with potential predictor variables were conducted, see Supplemental Table. At the individual-level potential predictors included father age, psychological distress and parenting self-efficacy. At the interpersonal level, predictors included coparenting, mother psychological distress, child sex, child expressive and receptive language ability, child social functioning, child behaviour and number of children in household. While at the social environment level, predictors included father language, indigenous status, education, employment, income, local area remoteness, socio-economic position (SEIFA) and stressful life events. Only variables with significant (*p* < .05) zero-order correlations with IPC for fathers of autistic children were entered into the regression models.

With respect to missing data, 23.3% was missing across all variables of interest. For Aim 3, missing data were handled using Full Information Maximum Likelihood (FIML) in MPlus. FIML is a commonly used missing data technique which uses all available data for individual cases to estimate model parameters (Little et al., 2014) and requires cases to have data on at least one variable of interest in the model to be estimated. For Aims 1, 2 and 4, missing data were handled using multiple imputation in SPSS. The results for analysis were pooled across 40 parallel imputed datasets incorporating variables that influence missing responses (i.e. number of children in household, financial hardship) along with all analysis variables. The pattern of results for cases with complete data and those with imputed data was similar, and therefore, only the results for cases with imputed data are reported.

## Results

### Estimates of IPC for fathers with autistic and non-autistic children

Table 3 presents the descriptive statistics for the total scale IPC scores at each timepoint for fathers of autistic children and fathers of non-autistic children. For both groups, IPC appeared to be low at each timepoint, with a slight decline over time. Without Bonferroni adjustment, fathers of autistic children were significantly more likely to report higher mean scores of IPC at child ages 4–5, 6–7, 8–9 and 10–11 years than fathers of non-autistic children. However, the effect sizes are small for each comparison. With Bonferroni adjustment, no significant differences in IPC scores over time were found between fathers of autistic children and fathers of non-autistic children. For both groups of fathers, reports of IPC mean scores appeared to be highest at child ages 4–5 years and lowest at 14–15 years. When examining differences in verbal and physical IPC, for both groups, verbal IPC was more commonly reported than physical IPC at each timepoint. For fathers of autistic children, the proportion of fathers reporting high verbal conflict peaked at child aged 8–9 years. Without Bonferroni adjustment, fathers of autistic children were also significantly more likely to report higher verbal IPC at this timepoint (8–9 years) than fathers of non-autistic children, again with a small effect size. With Bonferroni adjustment, no significant differences were found.

**Table 3.** Descriptive statistics for IPC scores at each wave for fathers of autistic children and fathers of non-autistic children.

Timepoint/child age	Fathers of autistic children (*n* = 281)			Fathers of non-autistic children (*n* = 7046)			*p*	*d*	High Verbal IPC, *n* (%)^a^				High Physical IPC, *n* (%)^a^	
	Range	*M*	*SD*	Range	*M*	*SD*			Fathers of autistic children (*n* = 281)	Fathers of non-autistic children (*n* = 7046)	*p*	Phi	Fathers of autistic children (*n* = 281)	Fathers of non-autistic children (*n* = 7046)
1. 4–5 years	3−19	9.83	2.76	2−25	9.56	2.51	.034*	−.106	22 (9.9)	554 (9.5)	.816	.002	1 (0.5)	48 (0.8)
2. 6–7 years	5−17	9.71	2.68	2−25	9.19	2.49	.038*	−.207	22 (10.7)	471 (6.7)	.382	.012	2 (0.7)	35 (0.5)
3. 8–9 years	5−19	9.57	2.72	4−25	9.14	2.48	.048*	−.174	30 (16.0)	437 (9.3)	.005**	.044	2 (0.7)	39 (0.6)
4. 10–11 years	5−18	9.34	2.67	1−25	9.05	2.49	.033*	−.115	21 (11.5)	409 (8.9)	.234	.017	1 (0.4)	36 (0.6)
5. 12–13 years	5−18	9.45	2.63	2−25	9.08	2.55	.321	−.147	20 (13.0)	389 (9.7)	.169	.021	0 (0.0)	28 (0.4)
6. 14–15 years	5−17	9.30	2.64	1−25	9.04	2.50	.335	−.104	19 (12.3)	348 (9.2)	.205	.020	2 (0.9)	27 (0.5)

a Sample size and denominator varied across waves due to missing data.

* Significant at *p* < .05; **significant at *p* < .01; Bonferroni adjusted α = .004.

### Trajectories of IPC for fathers with autistic and non-autistic children

To identify subgroups of fathers of autistic children defined by their trajectories of IPC over time (Aim 3), LLCA was conducted. Separate models were estimated for one to five classes (Table 3). After inspection of all model fit indices (Table 4), the three-class model was selected as the final model. The AIC and BIC for the three-class models were lower than those for the two-class models, suggesting improved model fit with the addition of the third class. While entropy for the three-class models dropped slightly compared to that for the two-class models, the three-class model still had high average posterior probabilities (Class 1 = 89.5%, Class 2 = 83.1%, Class 3 = 91.1%), thus indicating good accuracy in assigning fathers to their most likely class. The Vuong-Lo-Mendell-Rubin statistic indicated a significant difference between the two- and three-class models, suggesting that the three-class model gives a significant improvement in fit over the two-class model. LLCA was then conducted for fathers of non-autistic children so descriptive comparisons could be made (see Supplemental Model Fit Table). For fathers of non-autistic children, a two-class model best fit the data (*L*^2^ = −61,926.624, BIC = 124,021.592, AIC = 123,891.248, entropy = 0.751, Vuong-Lo-Mendell Rubin *p*-value < .001) with high posterior probabilities (Class 1 = 93.7%, Class 2 = 89.9%).

**Table 4.** Model fit indexes for latent classes of IPC for fathers of autistic children.

Model	*L* ^2^	BIC	AIC	Entropy	Vuong-Lo-Mendell-Rubin	*p*-value
1-Class	−2654.335	5376.331	5332.671	-	-	-
2-Class	−2464.715	5036.558	4967.430	0.838	1 vs 2 classes	<.0001
3-Class	−2399.988	4946.572	4851.975	0.746	2 vs 3 classes	.0001
4-Class	−2384.426	4954.918	4834.852	0.744	3 vs 4 classes	.2841
5-Class	−2373.883	4973.301	4827.767	0.771	4 vs 5 classes	.7593

**Figure 1.**
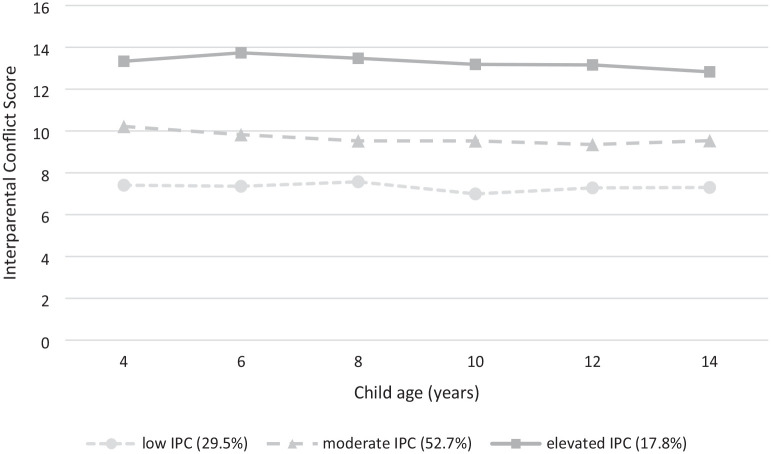
Trajectories of IPC across 10 years of child development for fathers of autistic children.

The trajectories of IPC over time for the three-class solutions for fathers of autistic children are presented in Figure 1. The first class represented fathers assigned to a trajectory of ‘*low and stable*’ reports of IPC across the 10 years of child development (*n* = 83, 29.5%). The second class represented fathers who experienced ‘*moderate and stable*’ levels of IPC (*n* = 148, 52.7%), while the final class represented a portion of fathers who experienced ‘*persistently elevated*’ levels of IPC over 10 years of child development (*n* = 50, 17.8%), see Table 5 for IPC descriptives across each timepoint for each class. Patterns of IPC over time for fathers of non-autistic children are presented in the Supplemental Tables, including a 2- and 3-class solution along with means and standard deviations for each group.

**Table 5.** Descriptive statistics for IPC across each class at each timepoint for fathers of autistic children.

Timepoint/child age	Class 1: Low IPC *n* = 83	Class 2: Moderate IPC *n* = 148	Class 3: Elevated IPC *n* = 50
	*M (SD)*	*M (SD)*	*M (SD)*
1. 4–5 years	7.59 (1.21)	10.23 (1.77)	12.27 (2.45)
2. 6–7 years	7.51 (1.21)	9.74 (1.50)	13.27 (2.00)
3. 8–9 years	7.35 (1.34)	9.48 (1.42)	13.55 (1.29)
4. 10–11 years	6.93 (1.33)	9.58 (1.67)	12.55 (0.93)
5. 12–13 years	7.21 (1.15)	9.65 (1.48)	13.09 (2.34)
6. 14–15 years	7.38 (1.68)	9.88 (1.81)	11.91 (1.87)

### Predictors of latent classes reflecting moderate and stable and persistently elevated IPC for fathers of autistic children

Table 6 presents the bivariate and multivariate logistic regression analyses to identify predictors of class allocation for fathers of autistic children. At the bivariate level, early childhood (timepoint 1, child aged 4–5 years) factors that were significantly associated with a ‘*moderate and stable*’ course of IPC included higher psychological distress reported by fathers and their partners, child behaviour difficulties, greater concerns with child receptive language and lower parenting self-efficacy and co-parenting support. Factors significantly associated with a course of ‘*persistently elevated*’ IPC at the bivariate level included increased father age, higher father and partner psychological distress, lower parenting self-efficacy, lower coparenting support and fathers living in regional or rural areas. The final multivariate model (Aim 4) revealed that compared with fathers in the ‘*low and stable*’ IPC group, fathers in the ‘*moderate and stable*’ IPC class had greater odds of reporting lower parenting self-efficacy and coparenting support and greater concerns with child receptive language. While factors for fathers in the ‘*persistently elevated*’ class in the final multivariate analysis included fathers being older, reporting lower coparenting support, higher partner psychological distress and higher parent reported child social functioning.

**Table 6.** Psychosocial factors for fathers of autistic children within the ‘moderate’ and ‘elevated’ IPC classes at child age 4–5 years.

	Class 1: Low IPC *n* = 83 M^a^	Class 2: Moderate IPC *n* = 148 M^a^	Class 3: Elevated IPC *n* = 50 M^a^	Moderate IPC OR (95% CI), *p*		Elevated IPC OR (95% CI), *p*	
				Univariate model	Multivariate model	Univariate model	Multivariate model
*Individual*							
Older father age	36.65	36.83	40.06	1.01 (.96–.1.05), .819	1.01 (.950–1.063), .862	1.10 (1.036–1.169), .002	1.15 (1.062–1.253), <.001
Higher father psychological distress	2.37	4.21	5.48	1.22 (1.077–1.374), <.002	1.12 (.969–1.294), .126	1.32 (1.146–1.527), <.001	1.17 (.984–1.398), .075
Higher father parenting self-efficacy	3.97	3.50	3.39	.505 (.342–.746), <.001	.562 (.351–.898), .016	.430 (.264–.699), <.001	.661 (.355–1.233), .193
*Interpersonal*							
Higher co-parenting support	4.50	4.19	3.90	.321 (.170–.605), <.001	.381 (.177–.823), .014	.125 (.052–.298), <.001	.150 (.050–.444), <.001
Higher partner psychological distress	3.21	4.69	5.11	1.144 (1.037–1.262), .007	1.116 (.998–1.248), .053	1.176 (1.050–1.317), .005	1.222 (1.059–1.411), .006
Higher child behaviour difficulties	34.12	35.63	35.27	1.097 (1.022–1.177), .011	1.049 (.961–1.145), .281	1.074 (.981–1.175), .125	1.085 (.953–1.234), .218
Higher child social functioning	71.10	67.89	76.56	.994 (.981–1.006), .326	1.014 (.996–1.033), .120	1.012 (.995–1.030), .170	1.040 (1.013–1.067), .003
*Child receptive language concern*							
Yes	21 (25.30%)	75 (50.68%)	13 (26.00%)	3.033 (1.681–5.475), <.001	3.943 (1.801–8.632), <.001	1.037 (.465–2.315), .929	1.934 (.659–5.676), .230
No	62 (74.70%)	73 (49.32%)	37 (53.62%)	Ref	Ref	Ref	Ref
*Socio-demographic*							
Remoteness							
Regional/Remote	38 (45.78%)	51 (34.46%)	10 (20.00%)	.623 (.360–1.078), .091	.741 (.379–1.450), .382	.296 (.131–.670), .003	.374 (.139–1.008), .052
Metro/Major city	45 (54.22%)	97 (65.54%)	40 (48.19%)	Ref	Ref	Ref	Ref
*Income*							
⩽$25,999	7.4 (8.92%)	15.6 (10.54%)	2.3 (4.60 5%)	.645 (.164–2.539), .531	1.107 (.202–6.073), .907	.202 (.029–1.407), .106	.478 (.041–5.637), .558
$26,000–$51,999	26 (31.33%)	45.3 (30.61%)	20.1 (40.2%)	.535 (.169–1.688), .286	.466 (.112–1.942), .294	.516 (.142–1.881), .316	.548 (.092–3.275), .509
$52,000–$103,999	44 (53.01%)	69.6 (47.02%)	19.5 (39.0%)	.487 (.163–1.460), .199	.369 (.094–1.458), .155	.297 (.083–1.058), .061	.240 (.043–1.327), .102
⩾$104,000	5.4 (6.51%)	17.5 (11.82%)	8.1 (16.2%)	Ref	Ref	Ref	Ref

a standard deviation not produced for MI pooled data; *p < .05; **p < .01; ***p < .001; *N* = 281; Low IPC as Reference Group.

## Discussion

This is the first longitudinal population-based study to explore reports of IPC across 10 years of child development for Australian fathers of autistic children. Fathers of autistic children reported relatively low levels of IPC across the 10-year period. Although small effect sizes, they were significantly more likely to report greater IPC than fathers of non-autistic children when children were aged 4–5, 6–7, 8–9 and 10–11 years. Notably, the highest levels of IPC, on average, were seen when children were aged 4–5 years, which aligns with the typical diagnostic period for Australian children (Bent et al., 2015; Gibbs et al., 2019). Many fathers report the diagnostic period as challenging, where they initially feel complex emotions such as shock and grief (Jagan & Sathiyaseelan, 2020) and undergo a period of adjustment and coming to terms with their family circumstances and their child’s needs (Brown et al., 2021). This is further compounded by fathers’ reports of a lack of accessible information about autism and feeling largely unsupported by health professionals during this time (Brown et al., 2021) and may indicate this time is particularly stressful for fathers and the couple’s relationship.

The lowest levels of conflict for fathers of autistic children were reported when children were aged 14–15 years. This may indicate potential improvement or adaptation in fathers’ overtime (D. Gray, 2002; Rattaz et al., 2023; Seymour et al., 2024). In terms of types of conflict, verbal conflict was more commonly reported than physical conflict at each timepoint. High verbal conflict was reported by 10%–16% of fathers of autistic children aged 4–14 years, where a peak in verbal conflict was noted when children were 8–9 years of age. High physical IPC was relatively uncommon (<1% at each timepoint), aligning with existing community-based research suggesting that conflict reported within couple’s relationships is typically of a verbal nature (Giallo, Seymour, Fogarty, et al., 2022; Westrupp et al., 2015).

In line with our first hypothesis, significant variability in fathers’ experiences of IPC over their parenting journey was found, with three distinct trajectories of IPC identified for fathers of autistic children. Approximately a third of fathers (29%) were classified into the ‘*low and stable*’ IPC trajectory, indicating consistently low levels of conflict over the 10-year period. A second group of fathers (53%) were classified into a ‘*moderate and stable*’ IPC trajectory. On average, IPC scores indicated that these fathers may be experiencing occasional but ongoing challenges in their relationship. Fathers in this group were more likely to endorse ‘*sometimes*’ to ‘*often*’ to verbal items including arguments with their partner and disagreements about child rearing; however, they were unlikely to report engaging in physical IPC behaviours. A final group of fathers (18%) were classified into the ‘*persistently elevated*’ IPC group, where IPC scores, on average, remained consistently high over the 10-year period. These fathers may be facing significant ongoing stress within the couple’s relationship and the wider family context, leading to heightened conflict. These fathers were significantly more likely to endorse ‘*sometimes*’ to ‘*always*’ having stressful, hostile conversations and arguments with their partner, along with endorsing ‘*rarely*’ to ‘*sometimes*’ engaging in physical IPC behaviours such as pushing and shoving. This suggests that some fathers of autistic children face enduring and heightened levels of IPC. Taken together, these findings emphasize fathers’ experiences of IPC vary considerably when raising an autistic child, with a significant proportion facing ongoing and elevated conflict across their parenting journey.

A range of individual, family, child and sociodemographic factors associated with trajectories of IPC for fathers of autistic children were explored to help improve early identification of IPC. These exploratory findings also highlight areas of future research into causal relationships to help inform intervention development. In line with our second hypothesis, a range of contextual factors were found to be associated with elevated IPC. Fathers within the ‘*moderate and stable*’ IPC class were more likely to report lower parenting self-efficacy and coparenting support and higher parent-reported child receptive language concerns than fathers in the low-IPC class. Conflict within intimate relationships is normal, and if resolved, constructively it can be healthy (Feeney, 2017). Within the current study, it was not possible to assess the mechanisms for conflict management and resolution, nor explore whether particular trajectories were associated with different communication and conflict resolution styles. Future research could explore whether there is a mediating relationship between parenting self-efficacy and co-parenting support and IPC, as these domains have been found to be associated with parental stress in parents of autistic children (May et al., 2015). It would also be beneficial for future research to explore the relationship between these trajectories and child and parent mental health outcomes to assess whether moderate levels of IPC can indicate healthy and positive adaptation within families with an autistic child.

Fathers in the ‘*persistently elevated*’ IPC group were found to have greater odds of being older, reporting lower levels of co-parenting support and having partners experiencing higher psychological distress along with children with higher parent-reported social functioning. As fathers age, they may face different stressors and challenges or have different coping mechanisms within the couple’s relationship. This is contrary to the general relationship literature which suggests that as partners age, they generally experience less conflict (Charles & Carstensen, 2010; Verstaen et al., 2020). Fathers within the ‘*persistently elevated*’ trajectory were also more likely to report lower coparenting support than fathers in the ‘*low and stable*’ IPC group. Indeed, lower levels of coparenting support have been associated with IPC in fathers in the general population (Kopystynska et al., 2022). Unexpectedly, higher parent-reported child social functioning (e.g. less problems with socializing, being bullied, playing with other children) was also associated with ‘*persistent and elevated*’ IPC trajectories. While higher parent-reported child social functioning is generally positive (Aishworiya et al., 2024; Wang et al., 2018), it may also bring about new challenges within the couple’s relationship (e.g. differing expectations). This is an important area for future research in understanding child functioning, family dynamics and the impact on parental conflict.

### Implications

The current findings have important implications for identification of IPC among fathers of autistic children. Importantly, the current findings highlight that there are different potential factors associated with different trajectories of IPC, suggesting that universal, one-size-fits-all approaches to reducing IPC are unlikely to be effective. Identifying fathers at risk of experiencing ‘*persistently elevated*’ IPC is crucial for timely support and preventing or minimizing the effects of IPC on parent, child and family wellbeing. Identification of fathers at risk of ‘*persistently elevated*’ IPC could be achieved through routinely asked questions within child-focused interventions. Targeted screening measures that assess relationship conflict, co-parenting support and parent mental health could be conducted early in the diagnostic/assessment process to identify these fathers and link them with additional support.

Targeted interventions focusing on improving coparenting support to reduce IPC experienced by fathers of autistic children could be further evaluated. Interventions and support that emphasize co-parenting support have been found to be beneficial in reducing conflict and improving mental health in parents of autistic children (Hock et al., 2022). Strengthening the couple’s ability to assist and guide one another through their parenting journey has the potential to aide in improved communication skills, parenting consistency and emotional support, thus positively impacting conflict resolution and contributing to healthier family dynamics (Cummings et al., 2001).

It is also important to support the whole family system when working with autistic children (Trembath et al., 2022). Fathers are often missed in services and support, despite identifying that they require additional support (Seymour et al., 2020, 2022). The current findings highlight that some fathers experience ongoing conflict within their a couple’s relationship. While interventions often focus on the child, recognizing the needs of all parents/care-givers and providing family-centred support can lead to more positive outcomes for the whole family (McCarthy & Guerin, 2022). This may align parents’ beliefs and expectations and promote cohesive parenting strategies.

### Limitations and future directions

There are several important limitations to consider when interpreting the current findings. These limitations also provide key directions for future research. First, inherent with longitudinal cohort studies, measures were necessarily brief and restricted. It was therefore not possible to assess the impact of other factors that may be associated with IPC such as conflict-management strategies, communication styles, social support, parenting stress and parent traits or diagnosis of neurodevelopmental conditions. The brevity of measures also extends to child diagnosis. The current study used primary caregiver reports of child diagnosis of autism. While parent reports have been found to be reliable and stable (May et al., 2021; Woolfenden et al., 2012), a comprehensive autism assessment would provide further insight into potential defining characteristics of autism, which may impact or be impacted by fathers’ reports of IPC. Furthermore, the relationship between IPC and some of the factors explored in the current analysis are likely to be complex, change over time and interrelated. Future research exploring bidirectional, mediating and moderating relationships is necessary to gain a better understanding of how to protect or buffer the effects of IPC on family wellbeing and guide intervention development. In addition, there is likely to be a level of bias in the current findings. Fathers who experience greater IPC are more likely to drop out or have missing data. Physical IPC reporting bias is also likely (e.g. not reporting physical IPC due to denial and/or shame). Therefore, the current results are likely to underreport the extent of IPC across time.

Second, mothers’ and fathers’ report of IPC were not compared. This is particularly important as IPC cannot occur in isolation. While a strength of the current study was the focus on fathers, future research comparing mothers’ and fathers’ experiences would contribute to a greater understanding of the complex interplay of IPC among families with autistic children and how this impacts parent and child outcomes. Research indicates that the experiences of mothers and fathers of autistic children differ (Greenlee et al., 2023; Jones et al., 2013) as do their support needs (Hartley & Schultz, 2015). Yet little is known about the similarities and differences of IPC experienced by mothers and fathers of autistic children and how best to support both parents so that family outcomes are maximized. It is important that interventions are cognizant and accommodating of these differences to increase efficacy and support the whole family. As such, future research exploring the interdependence of IPC in mothers and fathers of autistic children is needed to allow for within-couple differences to be identified (Hartley et al., 2011).

Finally, the study findings are limited in generalizability. Within the LSAC, there was an under-representation of single-parent and separated families, LGBTQIA+ families, families living in rental properties and fathers of Aboriginal or Torres Strait Islander and non-English speaking backgrounds. Further restricting generalizability, neurodivergence extends beyond autism, as such, further research is needed to explore a range of neurodevelopmental conditions (e.g. learning difficulties, attention-deficit hyperactivity) and the impact of co-occurring child conditions (e.g. anxiety, depression), along with diverse family backgrounds and compositions to assess whether the current trajectories of IPC can be extended to these fathers and their families.

## Conclusions

Crucial for effective identification, the results increase current understanding of challenges and needs faced by fathers of autistic children. The current study sheds light on the prevalence and trajectories of IPC for fathers of autistic children. IPC is complex, and the current study highlights that there is significant variation in the experiences of IPC among fathers of autistic children across child development. The current findings also contribute to our understanding of important potential factors associated with different patterns of IPC for fathers of autistic children. Interventions targeting modifiable psychosocial factors, such as parenting self-efficacy and coparenting support could have cascading effects on improving family dynamics and reducing IPC. Testing the efficacy of such interventions is an important area for future research, so fathers are provided with effective and targeted support. Future research is needed to further explore the longitudinal experiences of families raising autistic children, considering how the context of the couple’s relationship may impact child and parent mental health and wellbeing. By addressing partner conflict early, support can contribute to creating optimal family environments and improve family wellbeing. It is therefore essential that services consider the unique needs of fathers raising autistic children.

## Supplemental Material

sj-docx-1-aut-10.1177_13623613251316014 – Supplemental material for Psychosocial factors associated with the trajectories of interparental conflict for Australian fathers of autistic children: A longitudinal study across 10 years of child development Supplemental material, sj-docx-1-aut-10.1177_13623613251316014 for Psychosocial factors associated with the trajectories of interparental conflict for Australian fathers of autistic children: A longitudinal study across 10 years of child development by Monique Seymour, Grace McMahon, Ali Fogarty, Bridget O’Connor, Mark Feinberg, Rob Hock and Rebecca Giallo in Autism
