# Supplementary material for: Psychosocial factors associated with the trajectories of interparental conflict for Australian fathers of autistic children: A longitudinal study across 10 years of child development
Source: Autism. 2025 Feb 8;29(6):1554–68. doi: 10.1177/13623613251316014 (PMC12089675; doi:10.1177/13623613251316014)
Supplement: sj-docx-1-aut-10.1177_13623613251316014 – Supplemental material for Psychosocial factors associated with the trajectories of interparental conflict for Australian fathers of autistic children: A longitudinal study across 10 years of child development [file sj-docx-1-aut-10.1177_13623613251316014.docx]

Supplementary Table 1. Correlations between fathers’ IPC at each timepoint and potential predictor variables (at age 4-5yrs), pooled across 50 datasets.

| Predictor variables | IPC | | | | | |
| --- | --- | --- | --- | --- | --- | --- |
|  | 4-5  years | 6-7  years | 8-9  years | 10-11 years | 12-13 years | 14-15 years |
| *Individual* |  |  |  |  |  |  |
| Father age | .118 | .197** | .215** | .206** | .071 | .214** |
| Father psychological distress | .319*** | .290 *** | .167* | .163 | .151 | .152 |
| Father PSE | -.279*** | -.209** | -.108 | -.165* | -.097 | -.099 |
| *Interpersonal* |  |  |  |  |  |  |
| Coparenting | -.488*** | -.374** | -.276*** | -.301*** | -.290** | -.237** |
| Mothers’ K6 | .157* | .174* | .129 | .081 | .201* | .053 |
| Child sex | -.012 | -.099 | -.051 | -.075 | -.001 | -.043 |
| PEDS expressive lang concern | .124 | .072 | -.036 | .023 | -.032 | -.014 |
| PEDS receptive lang concern | .132* | .019 | -.021 | .070 | -.039 | -.046 |
| PEDS QL social func. | -.029 | .054 | .057 | .059 | .063 | .208* |
| P1 rated SDQ | -.157* | .057 | .082 | .150 | .101 | .077 |
| No. of children in household | .089 | .078 | .047 | .021 | -.001 | .009 |
| *Social environment* |  |  |  |  |  |  |
| Father language | .095 | -.035 | .085 | -.016 | .014 | .062 |
| Aboriginal or Torrer Strait Islander | -.092 | -.165* | -.012 | -.046 | -.111 | n/a |
| Education | -.024 | .042 | .104 | .039 | .031 | .156 |
| Employment | -.071 | -.077 | -.071 | -.135 | -.096 | -.054 |
| Income groups | .151* | .050 | .069 | .057 | .058 | .038 |
| Remoteness | -.193** | -.143* | -.131 | -.094 | -.131 | -.039 |
| SEIFA | -.004 | .012 | .009 | .009 | .010 | .017 |
| Stressful life events | .092 | .038 | .062 | .040 | .084 | -.050 |

** significant at p<.001; * significant at p<.05

Supplementary Table 2. Model fit indexes for latent classes of IPC for fathers of non-autistic children (n = 7046)

| Model | *L*^2^ | BIC | AIC | Entropy | Vuong-Lo-Mendell-Rubin | *p*-value |
| --- | --- | --- | --- | --- | --- | --- |
| 1-class | -65822.464 | 131751.250 | 131668.928 | - | - | - |
| 2-class | -61926.624 | 124021.592 | 123891.248 | 0.751 | 1 vs 2 classes | 0.0000 |
| 3-class | -60609.644 | 121449.654 | 121271.288 | 0.709 | 2 vs 3 classes | 0.0000 |
| 4-class | -60029.474 | 120351.335 | 120124.948 | 0.715 | 3 vs 4 classes | 0.0000 |
| 5-class | -59843.735 | 120041.879 | 119767.470 | 0.678 | 4 vs 5 classes | 0.3928 |

Supplementary Table 3. Descriptive statistics for IPC across each class at each timepoint for father of non-autistic children (n = 7046)

|  | 2-Class Model | |  | 3-Class Model | | |
| --- | --- | --- | --- | --- | --- | --- |
| Timepoint / child age | Class 1  n = 4933 (70%) | Class 2  n = 2113 (30%) |  | Class 1  *n* = 2898 (41%) | Class 2  *n* = 3341 (47%) | Class 3  *n* = 807 (12%) |
|  | *M (SD)* | *M (SD)* |  | *M (SD)* | *M (SD)* | *M (SD)* |
| 1. 4-5 yrs | 8.54 (1.76) | 12.06 (2.31) |  | 7.77 (1.55) | 10.28 (1.78) | 13.41 (2.45) |
| 2. 6-7 yrs | 8.14 (1.69) | 11.76 (2.27) |  | 7.36 (1.38) | 9.95 (1.68) | 13.25 (2.32) |
| 3. 8-9 yrs | 8.09 (1.68) | 11.66 (2.30) |  | 7.34 (1.42) | 9.83 (1.67) | 13.26 (2.33) |
| 4. 10-11 yrs | 8.01 (1.71) | 11.64 (2.21) |  | 7.22 (1.34) | 9.82 (1.68) | 13.09 (2.26) |
| 5. 12-13 yrs | 8.07 (1.79) | 11.63 (2.41) |  | 7.28 (1.46) | 9.92 (1.84) | 13.09 (2.59) |
| 6. 14-15 yrs | 8.05 (1.73) | 11.57 (2.41) |  | 7.36 (1.51) | 9.79 (1.77) | 13.08 (2.61) |
